# Disparities in objective sleep measures among individuals who have undergone polysomnographic studies

**DOI:** 10.3389/frsle.2025.1511969

**Published:** 2025-03-11

**Authors:** Min-Woong Sohn, Nathan A. Farr, Hyeeun K. Shin, Soojung Ahn, Myla D. Goldman, Sara N. Pasha, Hyojung Kang, Chae Won Kim, Jennifer M. Lobo

**Affiliations:** ^1^College of Public Health, University of Kentucky, Lexington, KY, United States; ^2^Cornell School of Nursing, Boston College, Chestnut Hill, MA, United States; ^3^Department of Neurology, School of Medicine, Virginia Commonwealth University, Richmond, VA, United States; ^4^Pulmonary, Critical Care and Sleep Medicine, College of Medicine, University of Kentucky, Lexington, KY, United States; ^5^College of Applied Health Sciences, University of Illinois at Urbana-Champaign, Champaign, IL, United States; ^6^School of Medicine, University of Virginia, Charlottesville, VA, United States

**Keywords:** sleep disparities, total sleep time, sleep disordered breathing, sleep quality, REM sleep time

## Abstract

**Background:**

Previous studies have amply demonstrated shorter self-reported sleep duration for Black patients compared to White patients. Evidence for disparities in objectively measured sleep is still mixed. Our objective is to assess disparities in objectively measured sleep parameters between races/ethnicities using data from an academic medical center in the US.

**Materials and methods:**

We used data extracted through text mining from sleep reports of in-lab polysomnography (PSG) studies conducted for adults aged 18 years or older at the University of Virginia Health System between 2010 and 2021. All studies with total sleep time (TST) <1 h were excluded. In multivariable analyses, we controlled for age, sex, body mass index, marital status, season, health insurance, comorbidities, and use of medications that may interfere with sleep in 1 year prior to the sleep study.

**Results:**

The study sample included 5,331 patients of whom 69% were non-Hispanic (NH) White, 23% NH Black, 4% Hispanic/Latinx, and 3% other or unknown. They were 57 ± 16, 52 ± 14, 49 ± 14, and 54 ± 14 years old and 45%, 30%, 33%, and 41% male, respectively. Average TST was 342 ± 84 min, sleep efficiency 74%, apnea-hypopnea index (AHI) 15 ± 17, with 69% having obstructive sleep apnea (36% mild; 22% moderate; 12% severe). After adjusting for covariates, Black patients had shorter N3 sleep by 10 min (95% CI = −11.9, −7.6) and longer REM sleep by 7 min (95% CI = 4.8, 8.6) compared to NH White patients. We did not find significant differences in TST, AHI, and sleep efficiency between racial/ethnic groups.

**Conclusions:**

Our objective data does not show consistently unfavorable sleep measures for racial and ethnic minorities. More research is needed to better understand how much of these differences are due to underlying physiology vs. social/environmental factors.

## Introduction

Racial and ethnic disparities in sleep are increasingly recognized as an important public health issue for their potential contribution to disparities in overall health, health outcomes, and mortality (Jackson et al., 2015; Billings et al., 2021; Khot et al., 2023; Denney et al., 2023). This concern was best conveyed by Grandner et al. (2016) who said “sleep disparities represent a pathway by which larger disparities emerge.” Sleep disparities are significant health issues by themselves but they are an even more critical problem seen as a driver of potentially much larger downstream health disparities.

Previous studies amply demonstrated that racial and ethnic minority people have shorter sleep time and poorer quality compared to White people (Ruiter et al., 2011; Grandner et al., 2013; Ahn et al., 2021). A recent study showed that the percentage of short sleepers (< 7 h of sleep) among African American or Black individuals was persistently higher than that among Caucasian or White individuals in a 15-year period between 2004 and 2018, with the gap between the two groups growing larger over time (Caraballo et al., 2022). However, much of the evidence on racial and ethnic disparities in sleep is based on subjective sleep and the lack of objective sleep data are often recognized as a limitation in this available research (Piccolo et al., 2013).

In our scoping review conducted in 2021 (Ahn et al., 2021), we found that the most consistent and significant finding in the sleep disparities literature was the disparities in subjective sleep duration experienced by Black people compared to White people. Data for Hispanic people or other racial and ethnic groups were much more limited and their results were mixed or insufficient. Importantly, the evidence on the racial/ethnic disparities in *objectively measured* sleep duration was mixed. In addition to sleep duration, other objective measures of sleep also were largely mixed or insufficient. To fill this gap in the literature, our aim is to examine sleep disparities between races and ethnicities using objectively-measured sleep data from a large academic medical center in the US.

## Methods

### Data sources and study sample

Medical records and sleep reports for persons who had received polysomnographic (PSG) sleep studies at the University of Virginia (UVA) Sleep Disorders Center in 2010–2021 were used in this study. Sleep reports were generated by either Sandman (until September, 2020) or Sleepworks software (since October 2020; both by Natus Medical Inc, Middleton, WI) from raw polysomnographic data. These reports are saved along with other medical notes as textual data in the electronic health records (EHR) system. We retrieved all sleep reports since the inception of the EHR at the UVA Health System (October, 2010) and extracted sleep values using text mining.

**Figure 1:**
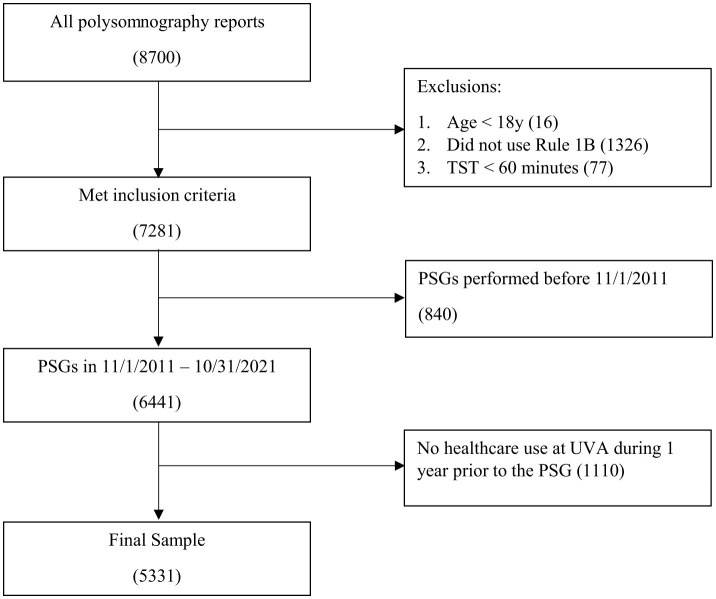
Sample flow diagram.

We included all studies conducted for adult patients aged 18 years old or older. We excluded from the sample all at-home sleep studies, split-night studies, and continuous positive airway pressure (CPAP) titration studies. For patients with multiple sleep studies, we utilized the first available study for analysis. Sleep studies with total sleep time < 60 min were excluded because their sleep measures including AHI (e.g., counts per hour) may be inaccurate. To reduce measurement errors, we only kept studies that used American Academy of Sleep Medicine Scoring rule 1B that require ≥4% oxygen desaturation when counting hypopneas (Berry et al., 2022; Grigg-Damberger, 2012). We also excluded studies performed before November, 2011 and studies pertaining to patients who did not utilize any healthcare services (outpatient visits, emergency room visits, or hospitalizations) during 1 year before the sleep study at the University of Virginia Health System in order to ensure that all patients have at least one full year of healthcare utilization data for identifying comorbidities. See Figure 1 for the sample flow diagram.

### Sleep measures

Sleep reports included TST, non-REM sleep (N1, N2, and N3 stages) and REM sleep times. Those times that were originally reported as percentages of TST were converted into durations in minutes. Sleep efficiency is the ratio of TST to time in bed expressed in percentages. AHI indicates total count of apneas and hypopneas per hour of sleep. Obstructive sleep apneas (OSA) severity was defined using AHI as normal (< 5), mild (5–14), moderate (15–29), and severe (≥ 30). Positional OSA (POSA) was identified as AHI ≥ 5 and supine and non-supine AHI ratio ≥ 2, while exclusive POSA (ePOSA) 5, 10, and 15 were identified as POSA plus non-supine AHI < 5, < 10, and < 15 per hour (Heinzer et al., 2015). We also extracted all other measures that are embedded within texts and tables on the sleep reports, including sleep latency, respiratory arousals, spontaneous arousals, periodic limb movements (PLMs), awakenings, and total stage changes. Sleep latency is defined as the time to the first sleep onset measured in minutes; respiratory arousals are the count per hour of brief awakenings from sleep due to breathing difficulties; spontaneous arousals are the count of brief awakenings without any obvious external stimulus; PLMs are involuntary, repetitive movements of the limbs during sleep and were counted separately for those associated with and those not associated with arousals; awakenings are the count of times when the patient wakes up during sleep.

### Race and ethnicity

Our study sample was divided into four groups based on their race and ethnicity (non-Hispanic White, non-Hispanic Black, Hispanic, and Other). We placed all patients who reported their ethnicity as Hispanic into one group, regardless of their race. Over 80% of all patients in the Other group did not report their race and the rest were Asians. We will henceforth use White and Black to refer to non-Hispanic White and non-Hispanic Black patients in this article.

### Covariates

Adjusted differences in sleep measures were estimated using multivariable models controlling for potential confounders that included age at the time of the sleep study, sex, body mass index (BMI), marital status (married or living with a significant other vs. not married or living alone), the season in which the sleep study was performed, Elixhauser comorbidities index (none, 1–2 conditions, 3–4 conditions, and 5 or more conditions) (Elixhauser et al., 1998), and prescriptions in the past 12 months of opioids, antipsychotics, antidepressants, and antiseizure medications that are known to affect sleep (Harrison et al., 2021; Seda et al., 2014). We included the season because OSA severity may vary significantly by season (Sevilla et al., 2023). Elixhauser comorbidities were determined using all diagnostic codes from healthcare encounters for 12 months prior to the sleep study. In all models other than the one predicting TST, we included TST as an additional covariate.

### Statistical analysis

**Figure 2:**
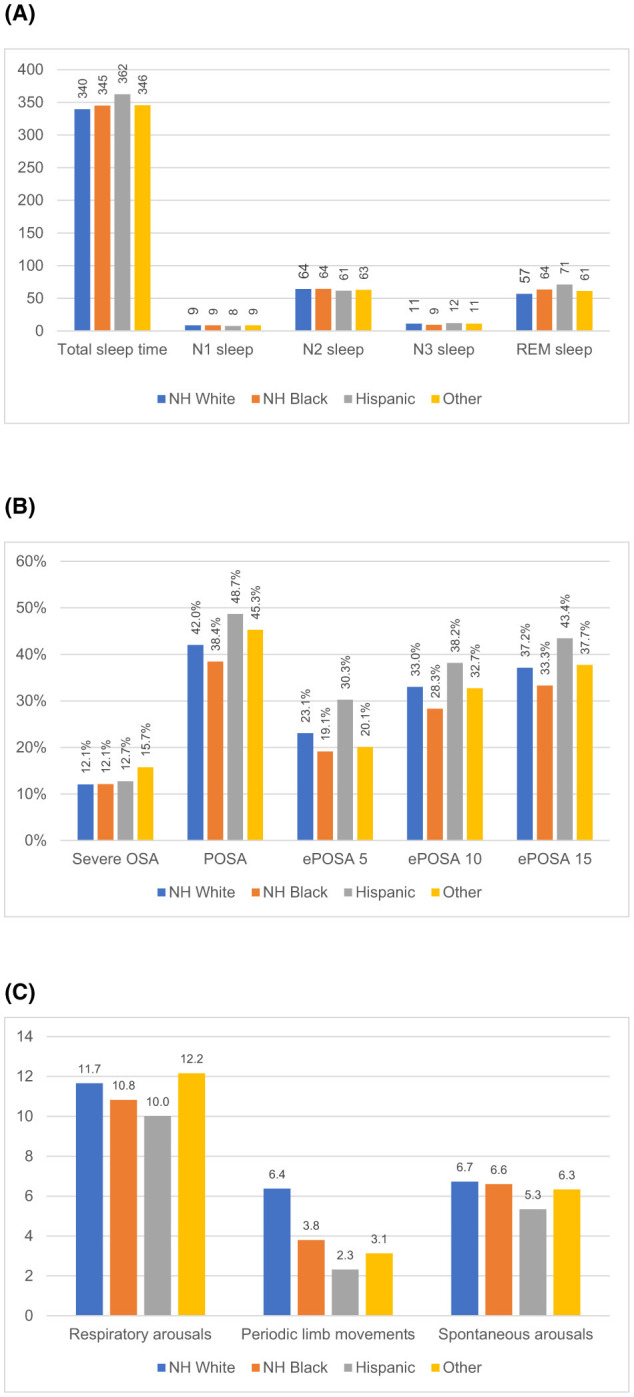
Unadjusted sleep measure by race and ethnicity. **(A)** Sleep time (minutes). **(B)** Sleep disordered breathing (percent of all patients). **(C)** Arousals (counts per hour).

**Figure 3:**
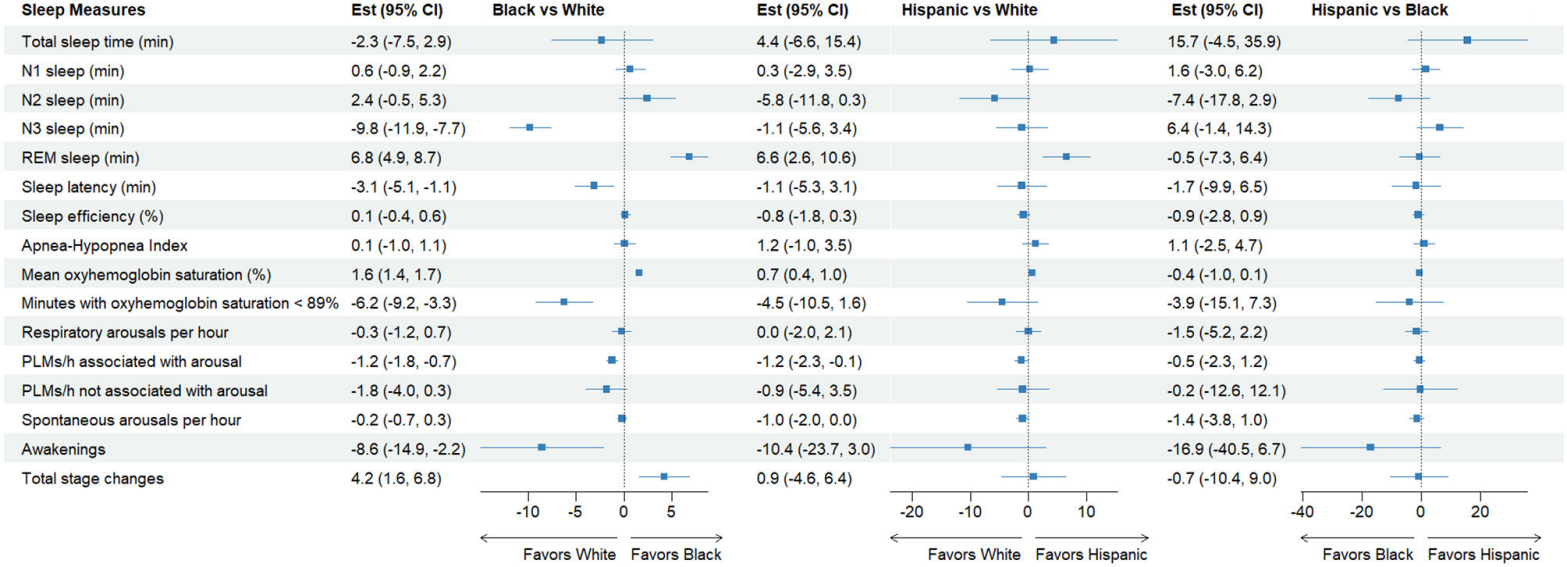
Forest plot for adjusted mean differences in select sleep measures. The model was adjusted for patient age, sex, body mass index, season of the sleep study, comorbidities, and medication use that affect sleep in the last 12 months. Total sleep time was also included in all models other than the one predicting total sleep time. The full model is shown in Table 3. REM, rapid eye movement; PLMs, periodic limb movements.

We compared patient characteristics and sleep measures by racial and ethnic groups using unadjusted bivariate analysis (Tables 1, 2, Figure 2). We then used multivariable linear regressions for continuous outcomes to estimate the mean differences for racial and ethnic minority groups compared to White patients (Table 3, Figure 3), and logistic regressions to estimate odds ratios for minority groups having a short-sleep (TST < 7 h), OSA (AHI ≥ 5), severe OSA (AHI ≥ 30), POSA, ePOSA-5, −10, and −15 compared to White patients (Table 4). Despite the fact that we are estimating 18 models in this study, we reported *p*-values without applying correction for multiple comparisons because our goal was to detect significant differences between groups and the correction would make it easier for us to reject the null hypotheses (Rothman, 1990; Gelman et al., 2012; Althouse, 2016). We also computed the Bonferroni-Holm corrected *p*-values and indicated statistically significant differences (*p* < 0.05) after the correction was applied with dagger symbols (^†^) in Tables 3, 4 (Holm, 1979). Adjusted mean differences and odds ratios were displayed in a forest plot for White-Black, White-Hispanic, and Black-Hispanic comparisons (Figure 2). This study was approved by the University of Virginia Institutional Review Board. We used Stata MP v18 (Stata Corp, College Station, TX) and Julia language v1.10 (MIT, Cambridge, MA) for statistical analysis.

**Table 1:** Patient characteristics by race/ethnicity.

**Variable**	**Race/ethnicity**					
	**All**	**NH White**	**NH Black**	**Hispanic**	**Other/Unknown**	***P*-value**
	***N* (%)**	***N* (%)**	***N* (%)**	***N* (%)**	***N* (%)**	
All, *n* (Row %)	5,331 (100.0%)	3,698 (69.37%)	1,246 (23.37%)	228 (4.28%)	159 (2.98%)	
Age at sleep study (y), mean (SD)	55.52 (15.21)	57.06 (15.53)	52.43 (13.78)	48.69 (13.54)	53.66 (14.31)	< 0.001
Male sex	2,179 (40.87%)	1,664 (45.00%)	375 (30.10%)	73 (32.02%)	67 (42.14%)	< 0.001
Married	2,434 (45.66%)	1,898 (51.33%)	352 (28.25%)	101 (44.30%)	83 (52.20%)	< 0.001
Body Mass Index (kg/m^2^), mean (SD)	34.72 (8.88)	33.84 (8.61)	37.66 (9.33)	34.82 (7.85)	32.15 (7.70)	< 0.001
**Health insurance**						
Commercial	1,203 (22.57%)	923 (24.96%)	223 (17.90%)	20 (8.77%)	37 (23.27%)	< 0.001
Medicare	2,156 (40.44%)	1,652 (44.67%)	450 (36.12%)	17 (7.46%)	37 (23.27%)	
Medicaid	683 (12.81%)	390 (10.55%)	242 (19.42%)	24 (10.53%)	27 (16.98%)	
Self-Pay	1,032 (19.36%)	550 (14.87%)	267 (21.43%)	162 (71.05%)	53 (33.33%)	
Other/Unknown	257 (4.82%)	183 (4.95%)	64 (5.14%)	5 (2.19%)	5 (3.14%)	
**Season of sleep study**						
Spring	1,347 (25.27%)	926 (25.04%)	316 (25.36%)	58 (25.44%)	47 (29.56%)	0.141
Summer	1,414 (26.52%)	987 (26.69%)	333 (26.73%)	66 (28.95%)	28 (17.61%)	
Fall	1,333 (25.00%)	939 (25.39%)	288 (23.11%)	62 (27.19%)	44 (27.67%)	
Winter	1,237 (23.20%)	846 (22.88%)	309 (24.80%)	42 (18.42%)	40 (25.16%)	
**Elixhauser comorbidity category**						
None	849 (15.93%)	614 (16.60%)	161 (12.92%)	34 (14.91%)	40 (25.16%)	< 0.001
1–2	2,290 (42.96%)	1,648 (44.56%)	446 (35.79%)	124 (54.39%)	72 (45.28%)	
3–4	1,605 (30.11%)	1,077 (29.12%)	437 (35.07%)	53 (23.25%)	38 (23.90%)	
≥5	587 (11.01%)	359 (9.71%)	202 (16.21%)	17 (7.46%)	9 (5.66%)	
Opioids	1,509 (28.31%)	999 (27.01%)	390 (31.30%)	69 (30.26%)	51 (32.08%)	0.018
Antipsychotics	254 (4.76%)	164 (4.43%)	67 (5.38%)	15 (6.58%)	8 (5.03%)	0.309
Antidepressants	326 (6.12%)	244 (6.60%)	70 (5.62%)	7 (3.07%)	5 (3.14%)	0.043
Antiseizure medication	372 (6.98%)	269 (7.27%)	81 (6.50%)	12 (5.26%)	10 (6.29%)	0.555

**Table 2:** Unadjusted differences in sleep measures by race/ethnicity (*N* = 5,331).^*^

**Variable**	**Race/ethnicity**					
	**All**	**NH White**	**NH Black**	**Hispanic**	**Other/Unknown**	***P*-value**
	***N* (%)**	***N* (%)**	***N* (%)**	***N* (%)**	***N* (%)**	
All, n (Row %)	5,331 (100.0%)	3,698 (69.37%)	1,246 (23.37%)	228 (4.28%)	159 (2.98%)	
TST (min), mean (SD)	342.00 (83.80)	339.59 (85.29)	344.98 (80.34)	362.42 (82.05)	345.52 (73.14)	< 0.001
TST < 7 h, mean (SD)	4,455 (83.57%)	3,106 (83.99%)	1,040 (83.47%)	174 (76.32%)	135 (84.91%)	0.024
N1 sleep (min), mean (SD)	26.84 (23.00)	27.02 (23.73)	26.44 (20.87)	25.46 (18.33)	27.76 (27.43)	0.645
N2 sleep (min), mean (SD)	218.68 (68.12)	217.09 (69.21)	222.75 (65.37)	223.32 (66.58)	217.33 (64.72)	0.055
N3 sleep (min), mean (SD)	37.32 (34.42)	38.62 (35.29)	32.27 (31.62)	42.56 (33.18)	39.29 (32.93)	< 0.001
REM sleep (min), mean (SD)	59.13 (36.14)	56.83 (36.38)	63.52 (34.66)	71.09 (38.81)	61.13 (31.30)	< 0.001
Apnea-Hypopnea Index (AHI), mean (SD)	14.68 (16.97)	14.66 (16.99)	14.62 (16.77)	14.40 (15.79)	15.84 (19.66)	0.844
**OSA severity**						
Normal	1,638 (30.73%)	1,136 (30.72%)	382 (30.66%)	67 (29.39%)	53 (33.33%)	0.946
Mild	1,894 (35.53%)	1,319 (35.67%)	441 (35.39%)	83 (36.40%)	51 (32.08%)	
Moderate	1,148 (21.53%)	797 (21.55%)	272 (21.83%)	49 (21.49%)	30 (18.87%)	
Severe	651 (12.21%)	446 (12.06%)	151 (12.12%)	29 (12.72%)	25 (15.72%)	
AHI in supine position, mean (SD)	22.92 (27.25)	23.36 (27.26)	21.11 (26.53)	23.71 (28.06)	25.67 (30.82)	0.041
AHI in non-supine position, mean (SD)	9.82 (15.55)	9.68 (15.54)	10.25 (15.36)	8.86 (14.36)	10.94 (18.70)	0.399
POSA	2,216 (41.57%)	1,554 (42.02%)	479 (38.44%)	111 (48.68%)	72 (45.28%)	0.012
ePOSA-5	1,192 (22.36%)	853 (23.07%)	238 (19.10%)	69 (30.26%)	32 (20.13%)	< 0.001
ePOSA-10	1,713 (32.13%)	1,221 (33.02%)	353 (28.33%)	87 (38.16%)	52 (32.70%)	0.004
ePOSA-15	1,948 (36.54%)	1,374 (37.16%)	415 (33.31%)	99 (43.42%)	60 (37.74%)	0.012
Sleep latency (min), mean (SD)	29.29 (32.82)	29.91 (32.88)	28.15 (33.33)	27.90 (33.05)	25.82 (26.37)	0.173
Sleep efficiency (%), mean (SD)	74.34 (15.89)	73.89 (16.08)	75.05 (15.57)	77.30 (15.58)	75.17 (13.66)	0.003
Mean oxyhemoglobin saturation (%), mean (SD)	94.20 (2.39)	93.77 (2.29)	95.31 (2.39)	94.85 (2.04)	94.53 (2.09)	< 0.001
Minutes with oxyhemoglobin saturation < 89%, mean (SD)	13.53 (37.18)	15.12 (39.43)	10.01 (30.27)	7.11 (17.18)	12.18 (46.57)	< 0.001
Respiratory arousals per hour, mean (SD)	11.41 (15.64)	11.66 (15.75)	10.83 (15.13)	10.01 (14.72)	12.16 (18.11)	0.184
PLMs/h associated with arousal, mean (SD)	2.26 (8.07)	2.71 (9.20)	1.34 (4.05)	1.05 (6.71)	0.88 (2.25)	< 0.001
PLMs/h not associated with arousal, mean (SD)	5.50 (32.05)	6.37 (18.25)	3.80 (57.96)	2.32 (11.56)	3.13 (11.55)	0.025
Spontaneous arousals per hour, mean (SD)	6.63 (7.39)	6.73 (7.86)	6.60 (6.42)	5.34 (4.40)	6.33 (6.38)	0.048
Awakenings, mean (SD)	106.60 (96.79)	109.95 (100.13)	99.44 (87.36)	94.79 (94.84)	101.64 (86.31)	0.002
Total stage changes, mean (SD)	85.86 (39.49)	85.26 (39.48)	87.22 (40.12)	83.86 (36.63)	91.88 (38.25)	0.085

^*^REM, rapid eye movement; OSA, obstructive sleep apnea; POSA, positional obstructive sleep apnea; ePOSA, exclusive positional sleep apnea; WASO, wakefulness after sleep onset; PLMs, periodic limb movements; NH, non-Hispanic.

**Table 3:** Mean differences in sleep measures for minority races/ethnicities compared to non-Hispanic White patients (*N* = 5,331).^*^

**Variable**	**NH Black**		**Hispanic**		**Other**	
	**Mean difference (95% CI)**	***P*-value**	**Mean difference (95% CI)**	***P*-value**	**Mean difference (95% CI)**	***P*-value**
TST (min)	−2.308 (−7.542, 2.927)	0.387	4.407 (−6.583, 15.397)	0.432	−3.503 (−16.047, 9.042)	0.584
N1 sleep (min)	0.642 (−0.877, 2.160)	0.407	0.298 (−2.890, 3.486)	0.855	1.797 (−1.842, 5.436)	0.333
N2 sleep (min)	2.398 (−0.490, 5.285)	0.104	−5.750 (−11.812, 0.311)	0.063	−1.779 (−8.698, 5.140)	0.614
N3 sleep (min)	−9.789 (−11.912, −7.666)	< 0.001^†^	−1.098 (−5.555, 3.359)	0.629	−0.998 (−6.086, 4.089)	0.701
REM sleep (min)	6.760 (4.861, 8.660)	< 0.001^†^	6.595 (2.607, 10.583)	0.001^†^	1.015 (−3.537, 5.566)	0.662
Sleep latency (min)	−3.110 (−5.121, −1.099)	0.002^†^	−1.097 (−5.320, 3.125)	0.610	−3.808 (−8.627, 1.011)	0.121
Sleep efficiency (%)	0.121 (−0.376, 0.619)	0.632	−0.787 (−1.831, 0.257)	0.140	0.078 (−1.114, 1.270)	0.898
Apnea-Hypopnea Index	0.059 (−1.012, 1.130)	0.914	1.247 (−1.001, 3.496)	0.277	2.331 (−0.235, 4.898)	0.075
AHI in supine position	−0.369 (−2.118, 1.379)	0.679	2.452 (−1.219, 6.123)	0.190	3.570 (−0.620, 7.760)	0.095
AHI in non-supine position	−0.011 (−1.000, 0.978)	0.983	0.170 (−1.906, 2.247)	0.872	2.337 (−0.033, 4.707)	0.053
Mean oxyhemoglobin saturation (%)	1.561 (1.417, 1.706)	< 0.001^†^	0.722 (0.419, 1.025)	< 0.001^†^	0.450 (0.104, 0.796)	0.011
Minutes with oxyhemoglobin saturation < 89%	−6.232 (−9.163, −3.302)	< 0.001^†^	−4.465 (−10.519, 1.589)	0.148	0.772 (−5.799, 7.343)	0.818
Respiratory arousals per hour	−0.260 (−1.255, 0.735)	0.609	0.042 (−2.047, 2.131)	0.968	1.374 (−1.010, 3.759)	0.259
PLMs/h associated with arousal	−1.239 (−1.775, −0.703)	< 0.001^†^	−1.214 (−2.339, −0.088)	0.035	−1.655 (−2.940, −0.370)	0.012
PLMs/h not associated with arousal	−1.844 (−3.971, 0.284)	0.089	−0.941 (−5.407, 3.525)	0.680	−1.897 (−6.995, 3.201)	0.466
Spontaneous arousals per hour	−0.206 (−0.697, 0.285)	0.410	−0.983 (−2.013, 0.048)	0.062	−0.370 (−1.546, 0.806)	0.538
Awakenings	−8.548 (−14.915, −2.181)	0.009	−10.371 (−23.738, 2.996)	0.128	−5.095 (−20.352, 10.163)	0.513
Total stage changes	4.188 (1.570, 6.807)	0.002^†^	0.911 (−4.586, 6.408)	0.745	7.027 (0.753, 13.302)	0.028

^*^Mean differences were computed from linear regressions that adjusted for the patient age at sleep study, sex, body mass index, season when the sleep study was conducted, Elixhauser comorbidities, use of opioids, antipsychotics, antidepressants, and antiseizure medications the one-year period before the sleep study. NH, non-Hispanic; TST, total sleep time; REM, rapid eye movement; AHI, Apnea-Hypopnea Index; PLM, periodic limb movement.^†^Indicates *p* < 0.05 after Bonferroni-Holm correction for multiple testing.

**Table 4:** Odds ratios and their 95% confidence intervals for obstructive sleep apnea indicators for minority races/ethnicities compared to non-Hispanic White patients (*n* = 5,331)^*^.

**Variable**	**NH Black**		**Hispanic**		**Other**	
	**OR (95% CI)**	***P*-value**	**OR (95% CI)**	***P*-value**	**OR (95% CI)**	***P*-value**
TST < 7 h	1.106 (0.920, 1.330)	0.283	0.866 (0.614, 1.222)	0.414	1.272 (0.801, 2.022)	0.308
OSA	1.003 (0.861, 1.168)	0.972	1.270 (0.917, 1.758)	0.150	1.054 (0.736, 1.510)	0.774
Severe OSA	1.065 (0.860, 1.321)	0.562	1.392 (0.897, 2.162)	0.141	1.659 (1.045, 2.631)	0.032
POSA	1.039 (0.903, 1.195)	0.594	1.490 (1.116, 1.988)	0.007^†^	1.238 (0.890, 1.723)	0.205
ePOSA−5	0.916 (0.774, 1.085)	0.309	1.617 (1.180, 2.216)	0.003^†^	0.866 (0.579, 1.295)	0.483
ePOSA−10	0.943 (0.813, 1.095)	0.443	1.448 (1.077, 1.947)	0.014	1.057 (0.747, 1.495)	0.754
ePOSA−15	1.003 (0.869, 1.157)	0.972	1.453 (1.087, 1.943)	0.012	1.098 (0.784, 1.538)	0.587

^*^Odds ratios were computed using logistic regression that adjusted for patient age at sleep study, sex, body mass index, season when the sleep study was conducted. NH, non-Hispanic; OSA, obstructive sleep apnea; POSA, positional sleep apnea; ePOSA, exclusive positional sleep apnea.^†^Indicates *p* < 0.05 after Bonferroni-Holm correction for multiple testing.

## Results

Our sample included 5,331 PSG studies conducted on the same number of patients who met our inclusion criteria. Patient characteristics by their race and ethnicity are shown in Table 1. Of all patients, 63% were White, 21% Black, 4% Hispanic, and 12% other or unknown race/ethnicity. The total patient population was 51 ± 14 years at the time of the PSG study, with Black and Hispanic patients about 5 and 8 years younger than White patients (*p* < 0.001). While the entire sample was comprised of 41% male, only 30% and 32% were male among Black and Hispanic patients (*p* < 0.001). While over 51% of White patients were married or living with a significant other, only 28% and 44% were among Black and Hispanic patients (*p* < 0.001).

Mean BMI for the sample was 34.7 ± 8.9 kg/m^2^ with Black patients having the highest BMI (37.7 ± 9.3 kg/m^2^) of all groups (*p* < 0.001). While 80% of Black patients were obese (BMI ≥ 30 kg/m^2^), only 62% and 71% were among White and Hispanic patients. Black patients also had the highest rates of Class III obesity (BMI ≥ 40 kg/m^2^) at 33% compared to ~20% for both White and Hispanic patients.

More than 84% of all patients had one or more co-existing conditions with over 40% having 3 or more. Other than obesity, the most prevalent comorbidity was hypertension (59%), followed by diabetes (35%), depression (25%), and chronic lung disease (23%). Of all racial/ethnic groups, Black patients had the highest prevalence of hypertension (74%), diabetes (69%), and chronic lung disease (25%), while White patients had the highest prevalence of depression (26%).

### Sleep time

The study patients slept an average of 342 min (5.7 h) in the lab with Hispanic patients having the longest (362 min) and White patients having the shortest TST (345 min). In adjusted models, we found small and non-significant differences in TST, which was ~2.4 min shorter for Black and Other patients and 4.8 min longer for Hispanic patients, compared to White patients (p > 0.05 for all comparisons).

Overall, 84% of the sample slept < 7 h. While 84% of White and 83% of Black patients had short sleep, only 76% did among Hispanic patients. Overall, the odds of having short sleep after adjusting for all covariates were not significantly different between White and all three minority groups.

These patients spent 26.8, 218.7, 37.3, and 59.1 min in N1, N2, N3, and REM sleep. While there were no significant differences in N1 sleep, there were significant unadjusted differences in N2, N3, and REM sleep by race/ethnicity. N3 sleep was the longest in Hispanic patients (43 ± 33 min), followed by patients in Other patients (39 ± 33 min) and White patients (39 ± 35 min). Black patients spent by far the shortest amount of time in N3 sleep (32 ± 32 min). In adjusted analysis, Black patients spent nearly 10 min shorter than White patients in N3 sleep (*p* < 0.001).

On the other hand, Hispanic patients had the longest REM sleep (71 ± 39 min), followed by Black patients (64 ± 35 min). White patients (57 ± 36 min) had the shortest REM sleep of all groups (*p* < 0.001). Black and Hispanic patients had 7 min longer REM sleep after adjusting for all covariates, compared to White patients (Table 3).

### Sleep disordered breathing

Mean AHI in the sample was 14.7 ± 17.0 but the median was 10 (interquartile range = 4–19), with no meaningful difference in AHI across groups. Almost 70% of all groups had OSA (AHI ≥ 5). The prevalence of POSA, ePOSA-5, -10, and -15 were all the highest among Hispanic patients and the lowest among Black patients. For example, POSA was found in 49% among Hispanic patients and in only 38% among Black patients. Similarly, ePOSA-5 was 30% and 19% in these two groups (*p* < 0.001 for both comparisons). In adjusted analyses, compared to White patients, Hispanic patients had 44%−61% higher odds of having these favorable breathing abnormalities but Black patients did not have any significant differences.

Mean oxyhemoglobin saturation was the highest among Black patients (95.3%) and the lowest among White patients (93.8%) (*p* < 0.001). The adjusted mean oxyhemoglobin saturation was 1.6% higher (95% CI = 1.4, 1.7; *p* < 0.001) for Black patients compared to White patients (*p* < 0.001). Likewise, the time spent with an oxygen saturation < 89% was the shortest for Hispanic patients (7.1 min) and the longest for White patients (15.1 min). The adjusted percentage of sleep time in oxygen saturation < 89% was about 6 min shorter (95% CI = −9.2, −3.3; *p* < 0.001) for Black patients compared White patients. Black and Hispanic patients were not significantly different from White patients.

### Sleep quality

Sleep efficiency was the highest among Hispanic patients at 77 ± 165% and the lowest among White patients at 74 ± 16% of all groups. But none was significantly different from other groups in adjusted models. Black patients had 3.1 min shorter latency (95% CI = −5.2, −1.1; *p* = 0.002) and 8.5 fewer awakenings per hour of sleep (95% CI = −14.9, −2.2; *p* = 0.009) but had 4.2 more stage changes (95% CI = 1.6, 6.8; *p* = 0.002) compared to White patients after adjusting for covariates, while patients in Hispanic and Other patients were not significantly different (Table 3).

### Arousals

In unadjusted data, respiratory arousals, periodic limb movements associated with arousals (PLMs), and spontaneous arousals were overall 11.4 ± 15.6, 2.3 ± 8.1, and 6.6 ± 7.4 per hour. In adjusted analyses, significant and substantial differences were found for PLMs with Black and Hispanic patients having about 1.2 fewer PLMs per hour compared to White patients (both *p* < 0.05). These represent over 40% fewer PLMs for Black and Hispanic patients compared to White patients.

## Discussion

Our results show that Black and Hispanic patients were not significantly different from White patients in most objective measures of sleep, including TST, AHI, or sleep efficiency. However, we found some interesting differences in sleep stage outcomes. In adjusted models, Black patients spent almost 10 min shorter in N3 (Slow Wave) sleep and spent 2 min longer in REM sleep than White patients. It is notable that both Black and Hispanic patients did not show differences in any of the measures of sleep disordered breathing, including AHI, OSA (AHI ≥ 5), severe OSA (AHI ≥ 30), POSA, ePOSA-5, -10, and -15. To the best of our knowledge, this study is the first that examined racial and ethnic disparities in positional OSA.

Overall, on most other measures that were significantly different in adjusted analyses, Black patients tended to have more favorable sleep compared to White patients, including shorter sleep latency, higher mean oxyhemoglobin saturation, shorter time spent in oxygen saturation < 89%, fewer PLMs, and awakenings. The only unfavorable sleep feature identified in Black patients was total stage changes compared to White patients. Hispanic patients show differences that are similar in both direction and magnitude of those in Black patients compared to White patients. However, their differences were mostly insignificant, perhaps due to their smaller sample size.

In the sleep disparities literature, the largest number of studies were conducted for racial disparities in subjective sleep duration, followed by subjective sleep quality/insomnia, and sleep disordered breathing (Ahn et al., 2022). Evidence so far overwhelmingly indicates that Black patients have shorter subjective sleep duration than White patients (Caraballo et al., 2022; Jackson et al., 2014; Cunningham et al., 2016; Seixas et al., 2019), but disparities in objective sleep duration is not so clear. A meta-analysis published in 2011 reported that, in eight studies of objective sleep duration, Black patients had 28 min (*p* < 0.05) shorter TST than White patients among normal sleepers (Ruiter et al., 2011). However, individual studies based on PSG reported mostly non-significant and inconsistent results (Redline et al., 1997; Rao et al., 1999; Irwin et al., 2000; Profant et al., 2002; Stepnowsky et al., 2003; Walsleben et al., 2004; Thomas et al., 2006; Mills et al., 2007; Mezick et al., 2008). It appears that the one actigraphy-based study had contributed a lion's share to the combined effect due to its large effect size as well as sample size (Lauderdale et al., 2006). Since then, a few additional studies have examined disparities in objectively measured sleep. Studies in which sleep duration was assessed using actigraphy showed relatively consistent findings favoring White patients (Mezick et al., 2008; Matthews et al., 2019; Song et al., 2011; Chen et al., 2015), while those assessed with PSG still showed mixed results (Hall et al., 2009; Fiorentino et al., 2006). It is our conjecture that the difference between actigraphy-based and PSG-based sleep time may derive from the sleeping environments at the time of measurement. For example, actigraphy studies are typically conducted at home and study subjects sleep in their usual sleeping environment, while in-lab PSG study subjects sleep in a highly uniform sleeping environment. Thus, actigraphy-measured sleep time is likely to also reflect differences in sleeping environments in terms of crowding, air quality, light, and noise at night (Jackson, 2017; Billings et al., 2019; Fang et al., 2015; Patel, 2019; Rudolph et al., 2019; Hammer et al., 2014).

Our results utilizing objective sleep duration are not consistent with most of the existing literature on disparities in subjective sleep duration. There are many reasons why objective and subjective sleep durations may be different, including the response bias inherent in self-reported values. Several studies (Takahashi et al., 2022; O'Donnell et al., 2009; Benz et al., 2023; Utsumi et al., 2022; Lauderdale et al., 2008) reported substantial differences between subjective and objective sleep durations which may be as large as 20% of TST which was driven by over-reporting among shorter sleepers (Lauderdale et al., 2008). One study even suggested that subjective and objective sleep durations may capture two different aspects of sleep (Benz et al., 2023). Another reason may be that our sample consisted of persons who had sleep complaints and were sleeping outside the usual sleeping environment with electroencephalogram (EEG) electrodes placed on the body. In addition, these are patients who had financial access to polysomnographic studies, either through their health insurance or self-payment (Stanchina, 2022). As such, our sample may represent a selective subset that may be different from those found in the communities. These are some of the explanations why racial/ethnic disparities based on subjective and objective sleep durations might diverge.

Another noteworthy finding in our study is the lack of disparities in most measures of sleep disordered breathing. Historically, evidence on racial and ethnic disparities in sleep disordered breathing in adults is largely mixed (Ahn et al., 2021; Dudley and Patel, 2016). Two recent studies illustrate the current state of evidence in this area. The first study based on data from an academic medical center in the US (Pranathiageswaran et al., 2013) showed a significantly higher AHI for the African American patients compared to White patients (median 32.7 vs 22.4; *p* = 0.01). The second study based on in-home PSG data for a subset of the Multi-Ethnic Study of Atherosclerosis (MESA) cohort (Chen et al., 2015) showed that Black participants were not significantly different from White participants in SDB severity, while Hispanic patients were. Our study based on in-lab PSGs showed no difference between groups in terms of AHI counts, prevalence of OSA or severe OSA (AHI ≥ 30), and measures of positional OSA between Black and White patients. On the other hand, Hispanic patients were doing significantly better than White patients in terms of all measures of positional OSA. While the first study counted hypopneas using the 3% desaturation rule, the MESA study and ours used the 4% desaturation rule. This might be one reason that can potentially explain the different results. Given that the first study and ours were based on in-lab PSGs, while the MESA study was based on in-home measures, the location does not seem to affect AHI measurement. More studies are needed if these two factors (hypopnea counting rule and measurement location) underlie the lack of clear evidence on racial and ethnic differences in sleep ordered breathing between racial and ethnic groups.

While the literature seems to offer insufficient or mixed results on racial and ethnic disparities in restorative sleep such as N3 and REM sleep (Ahn et al., 2021), our data show that Black patients had shorter N3 sleep but longer REM sleep, while Hispanic patients had longer N3 and REM sleep, compared to their White counterparts. Because these stages of sleep are linked to various health outcomes such as important for immune and autonomous nervous system and cardiovascular and brain health (N3) and emotional regulation, mortality and depression (REM) (Gottesman et al., 2024), there is a possibility that these differences can account for some part of the racial and ethnic disparities in these outcomes.

Our study has implications for clinical practice. First, sleep disparities are increasingly thought of as an important contributor to disparities in job performance, productivity, wages, and health. Recently, several investigators argued that sleep disparities may be the fundamental contributor to disparities in health outcomes, including cardiovascular disease, diabetes, and mortality (Jackson et al., 2015; Grandner et al., 2016; Kingsbury et al., 2013). And recommendations have been made to work on sleep as an intervention target for improved cardiovascular health (Jackson et al., 2015; Grandner et al., 2016; Kingsbury et al., 2013), obesity (Grandner, 2017), and disparities in overall health (Buxton and Marcelli, 2010). This is an important and even overdue development. However, from our vantage point after going through a large database of objective sleep measures, we feel that more studies are needed to confirm and understand disparities using objective sleep measures between races and ethnicities, especially because we did not find any significant disparities in sleep time and disordered breathing, two factors that are of paramount importance to cardiovascular disease (Jackson et al., 2015; Linz et al., 2015).

Second, there is a large number of sleep studies that are being done every year at many medical centers across the US. These studies are being recorded in raw data and sleep measures are being scored and pulled into either textual reports or computerized data fields in the electronic health records (EHR) systems. Combined with other data available from EHR systems such as diagnoses, procedures, lab results, vitals, and medication prescriptions, these real-life, objective data can be a large and valuable source of data that should be utilized more frequently in sleep research.

### Limitations

There are several limitations to consider when interpreting our results. First, our data were collected from individuals who sought and were referred for polysomnographic tests due to sleep complaints. Further, like any other polysomnographic studies, measures were taken from a single-night's sleep that occurred in a sleep laboratory with EEG sensors in place. Therefore, our data should be compared cautiously with measures taken from healthy individuals or from at-home studies. Second, these measures were taken from one institution that serves a large catchment area in the central Virginia in the US and therefore may have limited representativeness of average US adults with sleep issues. Third, study patients might have experienced disparities in access to healthcare and to polysomnographic studies. These disparities might have affected the data reported in this study. Fourth, we relied on electronic health records (EHRs) for patient comorbidities, races and ethnicities, and medications whose accuracy and completeness are unknown. Finally, EHRs also currently do not contain social determinants of health (SDOH) (Jackson, 2017; Grandner et al., 2015; Jean-Louis et al., 2022; Johnson et al., 2022; Rodrigues et al., 2024; Yoo et al., 2023) and we could not use them in this study. SDOH are important in sleep disparities and as such our findings should be interpreted with this limitation in mind.

### Conclusions

Our objective sleep data show that racial and ethnic minorities do not experience notable sleep disparities compared to White patients. We did not find significant differences in total sleep time, apnea and hypopnea index, and most measures of sleep quality and arousals. However, noteworthy differences included shorter N3 sleep time but longer REM sleep time for Black patients compared to White patients. Our study provides additional insights regarding racial/ethnic disparities in objective sleep to the growing literature in the field.

## Data Availability

The data analyzed in this study is subject to the following licenses/restrictions: The data underlying this article were provided by University of Virginia Health System by permission. In order for the data to be shared, a data use agreement and permission from the University of Virginia Health System would need to be in place. Requests to access these datasets should be directed to Jennifer M. Lobo, jem4yb@virginia.edu.
